# Deacetylation of metabolic enzymes by Sirt2 modulates pyruvate homeostasis to extend insect lifespan

**DOI:** 10.18632/aging.101447

**Published:** 2018-05-16

**Authors:** Tao Wang, Shao-Lei Geng, Yu-Min Guan, Wei-Hua Xu

**Affiliations:** 1 State Key Laboratory of Biocontrol, School of Life Sciences, Sun Yat-Sen University, Guangzhou 510006, China

**Keywords:** acetylation, sirtuin, glucose metabolism, diapause, *Helicoverpa armigera*

## Abstract

Diapause in insects is akin to dauer in *Caenorhabditis elegans* and hibernation in vertebrates. Diapause causes a profound extension of lifespan by low metabolic activity. However, the detailed regulatory mechanisms for low metabolic activity remain unknown. Here, we showed that low pyruvate levels are present in the brains of diapause-destined pupae of the cotton bollworm *Helicoverpa armigera*, and three enzymes pyruvate kinase (PK), phosphoenolpyruvate carboxykinase (PEPCK), and phosphoglycerate mutase (PGAM) are closely correlated with pyruvate homeostasis. Notably, Sirt2 can deacetylate the three enzymes to increase their activity *in vitro*. Thus, low Sirt2 expression in the brains of diapause individuals decreases PK and PEPCK protein levels as well as PGAM activity, resulting in low pyruvate levels and low tricarboxylic acid cycle activity and eventually inducing diapause initiation by low metabolic activity. These findings suggest that pyruvate is a checkpoint for development or lifespan extension, and Sirt2 is a negative regulator to extend lifespan in insects.

## Introduction

Most insect species have evolved a special stage of developmental arrest (diapause) in response to adverse environmental conditions [1]. Diapause, a specific entomological term, is used to describe the slow development as dauer in *Caenorhabditis elegans* [2] and hibernation in vertebrates [3] with low metabolic activity. For example, the cotton bollworm *Helicoverpa armigera* larvae are reared during periods of short day length and low temperatures (20 °C), and the pupae enter diapause with low metabolic activity [4]. Compared to their nondiapause counterparts, reared during periods of long day length under the same temperatures, the lifespan of diapause pupa is more than 3 times longer [4]. Therefore, this species is an excellent model for lifespan research [4].

Pyruvate can be produced from glucose and is then converted into acetyl-coenzyme A, which is the main input for a series of reactions, known as the tricarboxylic acid (TCA) cycle, to provide energy. Pyruvate is primarily produced by pyruvate kinase (PK), and two other enzymes, phosphoenolpyruvate carboxykinase (PEPCK) and phosphoglycerate mutase (PGAM), are closely correlated with pyruvate production. PK transfers a phosphate group from phosphoenolpyruvate (PEP) to ADP to produce pyruvate and ATP [5]. PGAM is a glycolytic enzyme that catalyzes the reversible conversion of 3-phosphoglycerate (3-PG) to 2-phosphoglycerate (2-PG), which is the precursor of phosphoenolpyruvate [6]. PEPCK is an enzyme that catalyzes oxaloacetate into phosphoenolpyruvate, a precursor pyruvate [7,8]. Thus, the three enzymes are involved in the regulation of pyruvate homeostasis.

A number of studies have shown that manipulating pyruvate homeostasis by transgene or dietary supplement can prolong or shorten the lifespan of animals. Supplementation with pyruvate at the *C. elegans* larval stage extends lifespan by increasing oxidative stress tolerance in a DAF-18/PTEN and Sir2.1-dependent manner [9]. During the larval period, the overexpression of malic enzyme, which oxidizes malate to pyruvate in the cytoplasm, lengthens the lifespan in *Drosophila melanogaster,* accompanied with increased ROS production and enhanced expression levels of ROS-scavenging enzymes [10]. The idea that increased pyruvate levels lengthen the lifespan is inconsistent with low metabolic activity, raising the question of whether such observations are experimental artifacts or operate at natural physiological levels. However, the inactivation of TCA cycle enzymes by RNAi promotes *C. elegans* longevity [11]; the reduced gene expression of *Indy* (the plasma membrane transporter for TCA intermediates) in *D. melanogaster* extends lifespan [12]; and the knockdown of homologs of *Indy* have also been related to extend lifespan in *C. elegans* and mice [13,14]. These results imply that low metabolic activity causes lifespan extension through decreased TCA cycle activity.

Protein acetylation is an important post-translational modification to regulate enzyme activity in sugar metabolism *via* acetyltransferases and deacetylases [15], and most acetylated metabolic enzymes undergo inactivity or degradation [16]. Previous studies have indicated that sustained high levels of protein acetylation might restrict lifespan [17], and sirtuin, including Sir2 in yeast, Sir2.1-2.4 in *C. elegans*, and Sirt1, 2, 4, 6, and 7 in *D. melanogaster*, is evolutionarily conserved NAD^+^-dependent deacetylase as anti-aging proteins [18]. Sir2 can extend the lifespan in yeast [19]. Overexpression of Sir2.1 increases lifespan in *C. elegans* via the insulin/IGF pathway transcription factor DAF-16/FoxO [20]. The life-extending effect of calorie restriction on aging in *D. melanogaster* has also been reported as Sirt1 dependent [21].

Despite extensive studies on the aging-related function of Sir2, little is known about the effect of Sirt2 on lifespan. Sirt2 is up-regulated during calorie restriction among various species [22,23]. One report shows that Sirt2 overexpression can increase lifespan by inducing checkpoint kinase BubR1 in mice [24]. Since Sirt2 also participates in the regulation of metabolically relevant progress, such as glycolysis, gluconeogenesis, and the pentose phosphate pathway [25–27], it is likely that Sirt2 may be related to lifespan through other pathways.

In the present study, we find that low levels of Sirt2 are present in brains of diapause-destined pupae to decrease PK and PEPCK protein levels as well as PGAM activity. The low activity of metabolic enzymes results in low levels of pyruvate, which are present under natural physiological conditions to profoundly extend lifespan by reducing TCA cycle activity in *H. armigera*. We reveal a novel connection between enzyme acetylation and lifespan extension in a Sirt2-dependent manner. This report is the first to show that Sirt2 is a negative regulator of lifespan, protein acetylation has a positive effect on lifespan by inhibiting glucose metabolism, and low physiological levels of pyruvate contribute to lifespan extension in insects.

## RESULTS

### Low pyruvate levels are essential for lifespan extension in *H. armigera*

**Figure 1:**
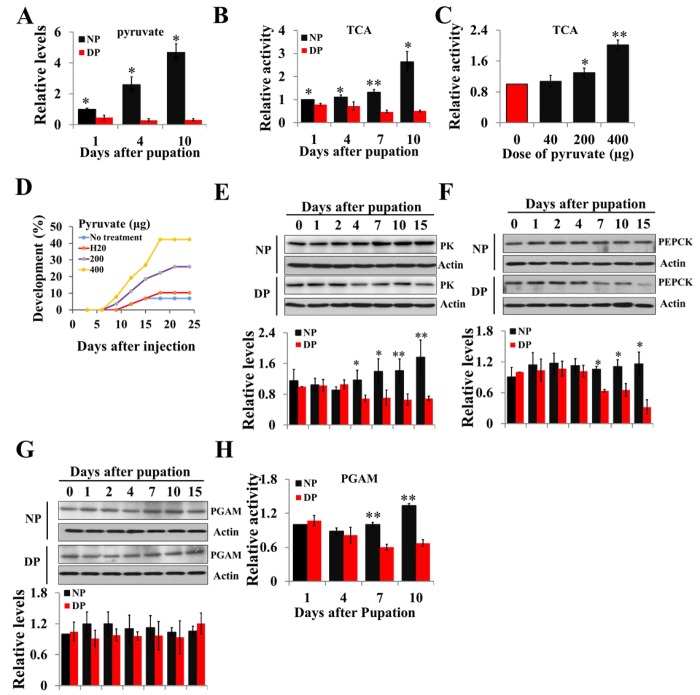
**Changes of pyruvate content, TCA activity, and expression patterns of PK, PEPCK, and PGAM during developmental pupal brain.** (**A**) Changes of pyruvate levels in brains of diapause- and nondiapause-destined pupae. (**B**) Changes of TCA cycle activity in brains of diapause- and nondiapause-destined pupae. (**C**) Changes in TCA cycle activity by injection of pyruvate. Day-1 diapause-destined pupae were injected with pyruvate and pupal brains were dissected 48 h after injection. Proteins from pupal were extracted and TCA activity was measured and normalized against total protein levels. (**D**) Termination of pupal diapause in response to pyruvate injection. Day-1 diapause-destined pupae were injected with pyruvate. No treatment, n=58; H_2_O, n=58; 200 μg, n=54; 400 μg, n=52. Pupae development was determined by examining the location of the pupal stemmata. Developmental expression of PK (**E**), PEPCK (**F**), and PGAM (**G**) by western blotting. Proteins from pupal brains were extracted and detected with corresponding antibodies. The bands were quantified using Image software (ImageJ) and normalized to the levels of *H. armigera* actin (5 μg). (**H**) PGAM activity in pupal brains. Enzyme activity was measured and normalized against total protein levels. DP, diapause-destined pupae; NP, nondiapause-destined pupae. Each point represents the means±S.D. of three independent replicates. *, p<0.05; **, p<0.01 (determined by independent t-test).

To confirm the different pyruvate levels and metabolic activity between nondiapause- and diapause-destined individuals, pupal brains were dissected, and pyruvate levels and TCA cycle activity determined by citrate synthase activity were measured. The results revealed that both pyruvate levels and TCA cycle activity are significantly greater in the brains of nondiapause-destined pupae than in those of diapause-destined pupae from day-1 after pupation (Figs. 1A and 1B), suggesting that low levels of pyruvate result in low metabolic activity, which leads to diapause initiation, and pyruvate may be used as a central component of the signaling system to monitor the development or diapause. To examine this idea, pyruvate was injected into diapausing pupae, and the results showed that TCA cycle activity increased while diapause was terminated (Figs. 1C and 1D). These results suggest that high levels of pyruvate and TCA cycle activity promote growth and development, whereas low levels of pyruvate and TCA cycle activity induce lifespan extension.

There are three metabolic enzymes—PK, PGAM and PEPCK—related to pyruvate homeostasis; we cloned the three cDNAs from *H. armigera* pupal brains by degenerate primers and RACE (rapid amplification of cDNA ends) strategy (Figs. S1-S3) and demonstrated that the amino acid sequences of the three proteins show high identity to those in mammals. The synthetic peptides, corresponding to the amino acid sequences of PK, PEPCK, and PGAM in humans, are used as antigens to produce polyclonal antibodies (PK and PEPCK from Abcam, PGAM from Santa Cruz). These synthetic peptide sequences show identity to those of *H. armigera* (Figs. S1-S3), and antibodies were purchased to investigate the expression of these three gene in insects by western blotting.

Diapause-destined pupae enter diapause 8-10 days after pupation; we focus on the diapause initiation phase from day-0 to day-15. The expression pattern of PK protein in the brains of diapause-destined pupae was similar to that of nondiapause pupae from day-0 to day-2 but significantly lower in diapause-destined pupae from day-4 to day-15 (Fig. 1E). The expression of PEPCK protein was similar in the brains of two pupae types from day-0 to day-4 but decreased in brains of diapause-destined pupae from day-7 to day-15, consistent with that of PK (Fig. 1F). PGAM protein levels showed no difference in the brains of two pupae types (Fig. 1G), but PGAM activity significantly decreased from day-7 to day-10 in diapause-destined pupae (Fig. 1H). These results indicate that low expression or low activity of the three enzymes is related to pyruvate biosynthesis, resulting in low pyruvate levels in diapause-destined pupae to induce lifespan extension or diapause via low metabolic activity.

### Three metabolic enzymes are negatively regulated by acetylation modification and the developmental expression of Sirt2

**Figure 2:**
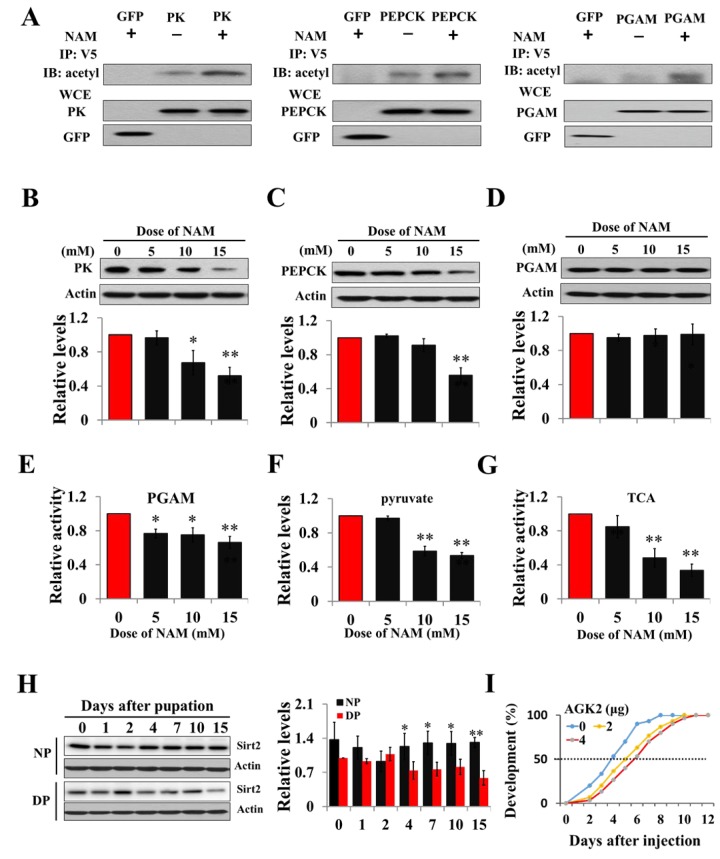
**Acetylation negatively regulates PK, PEPCK protein levels and PGAM activity**. (**A**) Acetylation analyses of PK, PGAM and PEPCK. GFP-PK-V5, GFP-PGAM-V5 or GFP-PEPCK-V5 plasmid was transfected into HzAm1 cells, followed by Sirt2 inhibitor NAM treatment. Protein acetylation was analyzed by immunoprecipitation (IP) and western blotting with anti-acetyl-lysine antibody. WCE: whole cell extracts. Changes in PK (**B**), PEPCK (**C**), and PGAM (**D**) protein levels in response to NAM treatment. (**E**) Changes in PGAM activity with NAM treatment. (**F**) Changes in pyruvate levels with NAM treatment. (**G**) Changes in TCA cycle activity with NAM treatment. HzAm1 cells were cultured in 0, 5, 10, and 15 mM NAM for 48 h. (**H**) Expression pattern of Sirt2 during pupal development. Protein extracts from the brains or cells were used for western blotting with the indicated antibodies. Protein bands were quantified using ImageJ software and normalized to the levels of *H. armigera* actin (5 μg). Pyruvate levels and enzyme activity was measured and normalized against total protein levels. DP, diapause-destined pupae; NP, nondiapause-destined pupae. **(I**) Developmental delay caused by AGK2 injection. Day-1 nondiapause-destined pupae were injected with 2 μg (n=60) or 4 μg (n=60) AGK2 or DMSO (n=60) as the control. Developmental delay was determined by examining the location of the pupal stemmata. Each point represents the means±S.D. of three independent replicates. *, p<0.05; **, p<0.01 (determined by independent t-test).

We speculated that acetylation might be present in the regulation of PK, PGAM, and PEPCK activity in insects. To examine this idea, recombinant PK-V5 or PGAM-V5 or PEPCK-V5 was transfected into HzAm1 cells, and the cell extracts were immunoprecipitated with an anti-V5 antibody. Western blotting with anti-acetyl-lysine antibody confirmed that the three proteins are acetylated and that acetylation levels are enhanced when treated with a broad range of deacetylation inhibitor nicotinamide (NAM) for Sirt family deacetylases (Fig. 2A). We further analyzed the structural character of three enzymes using PSKAcePred, a lysine acetylation prediction tool [28]. The potential acetylation sites were at K310 of PK, K100 of PGAM, and K95 of PEPCK, compared with known acetylation sites of their homolog proteins in mammals (Figs. S4A and S4B). To determine the effect of acetylation on PK, PGAM, and PEPECK, HzAm1 cells were treated with NAM, and the protein levels were detected by western blotting. Steady-state levels of endogenous PK and PEPCK were decreased by inhibitor (Figs. 2B and 2C), while the mRNA levels of these genes remained unchanged (Figs. S5A and S5B). Although the protein levels of PGAM were unchanged by NAM treatment, the activity of this protein was significantly decreased (Figs. 2D and 2E). Further, HzAm1 cells were treated with NAM, and pyruvate levels and TCA cycle activity also remarkably decreased (Figs. 2F and 2G), indicating that acetylation may lead to protein degradation or low enzyme activity and that these proteins might be regulated by the NAD^+^-dependent sirtuin family.

Previous studies have demonstrated that Sirt2 is involved in regulating sugar metabolism in mice [29,30], and PGAM and PEPCK are the substrates of Sirt2 [26,31]. We thus examined the feasibility of Sirt2 as the deacetylase for PK, PGAM, and PEPECK. The Sirt2 gene was cloned by degenerate primers and RACE (rapid amplification of cDNA ends) strategy (Fig. S6), and polyclonal antibody was made to investigate the expression pattern of Sirt2 in the brains of diapause- and nondiapause-destined pupae by western blotting. The results revealed that Sirt2 protein levels in diapause-destined pupae were similar to those of nondiapause pupae from day-0 to day-2 but significantly decreased in diapause-destined pupae from day-4 to day-15, consistent with PK and PEPCK expression (Fig. 2H) and indicating that low Sirt2 may participate in diapause initiation through the regulation of related metabolic enzymes.

To confirm Sirt2 function in diapause through the deacetylation of three metabolic enzymes, a Sirt2-selective inhibitor AGK2 was injected into day-1 nondiapause-destined pupae, and a developmental delay of approximately 2 days was observed based on pupal stemmata migration (Figs. 2I), suggesting that Sirt2 may be a key factor in diapause entry by regulating the three enzymes related to pyruvate homeostasis.

### Sirt2 interacts with and increases PK protein levels

**Figure 3:**
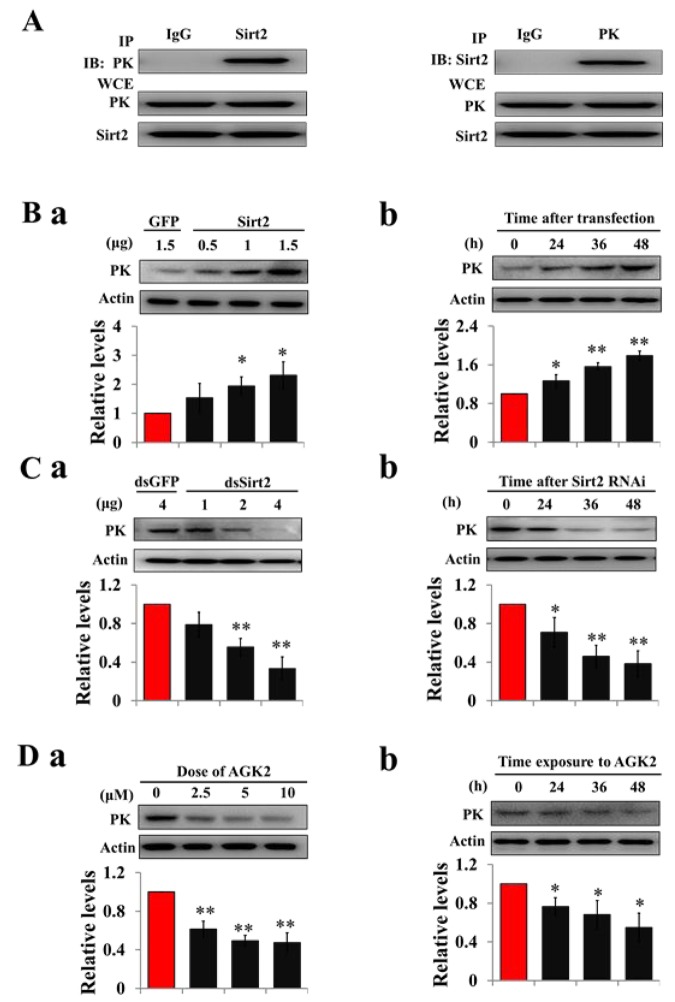
**Sirt2 interacts with and increases PK protein levels.** (**A**) Sirt2 physically interacts with PK. HzAm1 cells were co-transfected with GFP-Sirt2 and GFP-PK-V5 plasmid for 48 h, and then the cell extracts were immunoprecipitated (IP) with anti-V5 or anti-Sirt2 antibody, followed by immunoblotting (IB) with anti-Sirt2 or anti-V5 antibody, respectively. WCE: whole cell extracts. (**B**) Sirt2 transfection increases endogenous PK protein levels *in vitro*. (**a**) Dose-dependent response to Sirt2 transfection. HzAm1 cells were transfected with GFP-Sirt2 or GFP-V5 plasmid for 48 h. (**b**) Time-dependent response to Sirt2 transfection. HzAm1 cells were transfected with 1.5 μg Sirt2 plasmid. (**C**) Sirt2 knockdown decreases endogenous PK protein levels *in vitro*. (**a**) Dose-dependent response to Sirt2 RNAi. HzAm cells were transfected with Sirt2 dsRNA or GFP dsRNA for 48 h. (**b**) Time-dependent response to RNAi. HzAm1 cells were transfected with 4 μg Sirt2 dsRNA. (**D**) Effects of Sirt2 inhibitor AGK2 on PK protein levels. (**a**) Dose-dependent response to AGK2 treatment. HzAm1 cells were cultured with AGK2 for 48 h. (**b**) Time-dependent response to AGK2 treatment. HzAm cells were cultured with 10 μM AGK2. Proteins were extracted from the cells for immunoblotting with anti-PK antibody. Protein bands were quantified using ImageJ software and normalized to the levels of *H. armigera* actin (5 μg). Each point represents the means±S.D. of three independent replicates. *, p<0.05; **, p<0.01 (determined by independent t-test).

To determine whether deacetylase is responsible for PK deacetylation, recombinant Sirt2 was co-transfected with PK into HzAm1 cells, and co-immunopreciptiation showed that Sirt2 binds strongly to the PK, suggesting a potential role for Sirt2 in PK deacetylation (Fig. 3A). We then overexpressed Sirt2 in HzAm1 cells and detected PK expression by western blotting, showing that PK protein levels significantly increased in dose- and time-dependent manner (Fig. 3B). Furthermore, we applied dsRNA against Sirt2 to treat HzAm1 cells and found that down-regulation of Sirt2 results in a decrease of PK level (Figs. 3C and Fig. S7). The application of the selective Sirt2 inhibitor AGK2 [32] also decreased PK levels in HzAm1 cells (Fig. 3D). This evidence suggests that Sirt2 is responsible for PK deacetylation to regulate its protein levels.

### Sirt2 binds to and activates PGAM activity

**Figure 4:**
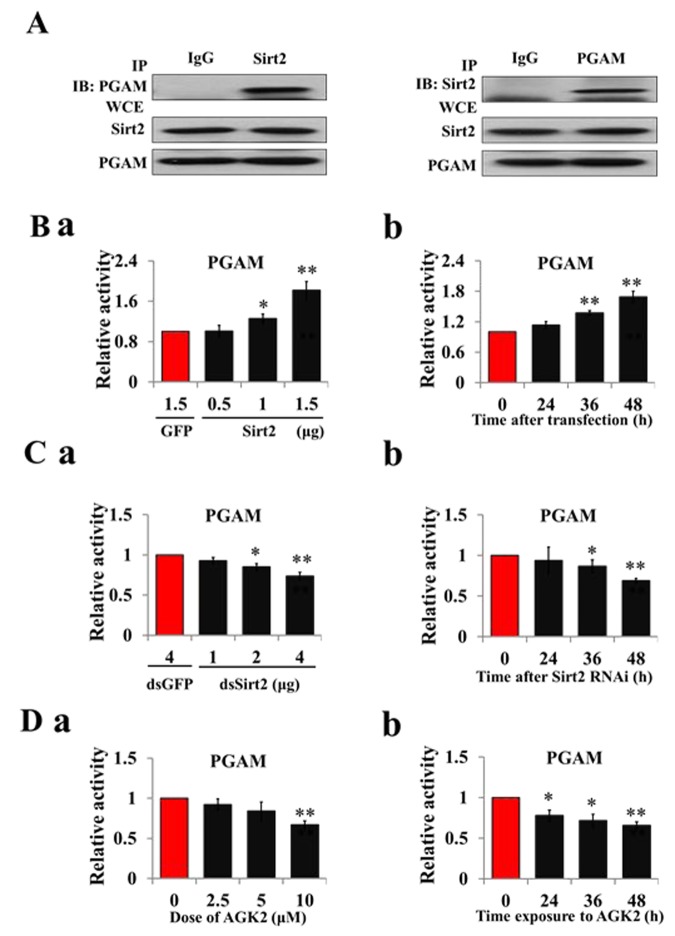
**Sirt2 interacts with and increases PGAM activity.** (**A**) Sirt2 physically associates with PGAM. HzAm1 cells were co-transfected with GFP-Sirt2 and GFP-PGAM-V5 plasmids for 48 h, and then the cell extracts were immunoprecipitated (IP) with anti-V5 or anti-Sirt2 antibody, followed by immunoblotting (IB) with an anti-Sirt2 or anti-V5 antibody, respectively. WCE: whole cell extracts. (**B**) Sirt2 overexpression increases PGAM activity *in vitro*. (**a**) Dose-dependent response to Sirt2 overexpression. HzAm1 cells were transfected with GFP-Sirt2 or GFP-V5 plasmid for 48 h. (**b**) Time-dependent response to Sirt2 transfection. HzAm1 cells were transfected with 1.5 μg GFP-Sirt2 plasmid. (**C**) Sirt2 knockdown decreases PGAM activity *in vitro*. (**a**) Dose-dependent response to Sirt2 RNAi. HzAm1 cells were transfected with Sirt2 dsRNA or GFP dsRNA for 48 h. (**b**) Time-dependent response to RNAi. HzAm1 cells were transfected with 4 μg Sirt2 dsRNA. (**D**) Effects of Sirt2 inhibitor AGK2 on PGAM activity. (**a**) Dose-dependent response to AGK2 treatment. HzAm1 cells were cultured with AGK2 for 48 h. (**b**) Time-dependent response to AGK2 treatment. HzAm1 cells were cultured with 10 μM AGK2. Enzyme activity was measured and normalized against total protein levels. Each point represents the means±S.D. of three independent replicates. *, p<0.05; **, p<0.01 (determined by independent t-test).

To test Sirt2 PGAM-modifying activity, recombinant Sirt2 was co-expressed with PGAM in HzAm1 cells, and co-immunoprecipitiation showed that Sirt2 and PGAM bind to each other (Fig. 4A). Sirt2 overexpression increased PGAM activity (Fig. 4B) and Sirt2 knockdown by RNAi decreased PGAM activity in HzAm1 cells in dose- and time-dependent manners (Fig. 4C). Furthermore, cells treated with Sirt2 inhibitor AGK2 also showed decreased PGAM activity (Fig. 4D). These results indicate that Sirt2 physically interacts with PGAM and increases its activity by deacetylation.

### Sirt2 interacts with and increases PEPCK protein levels

**Figure 5:**
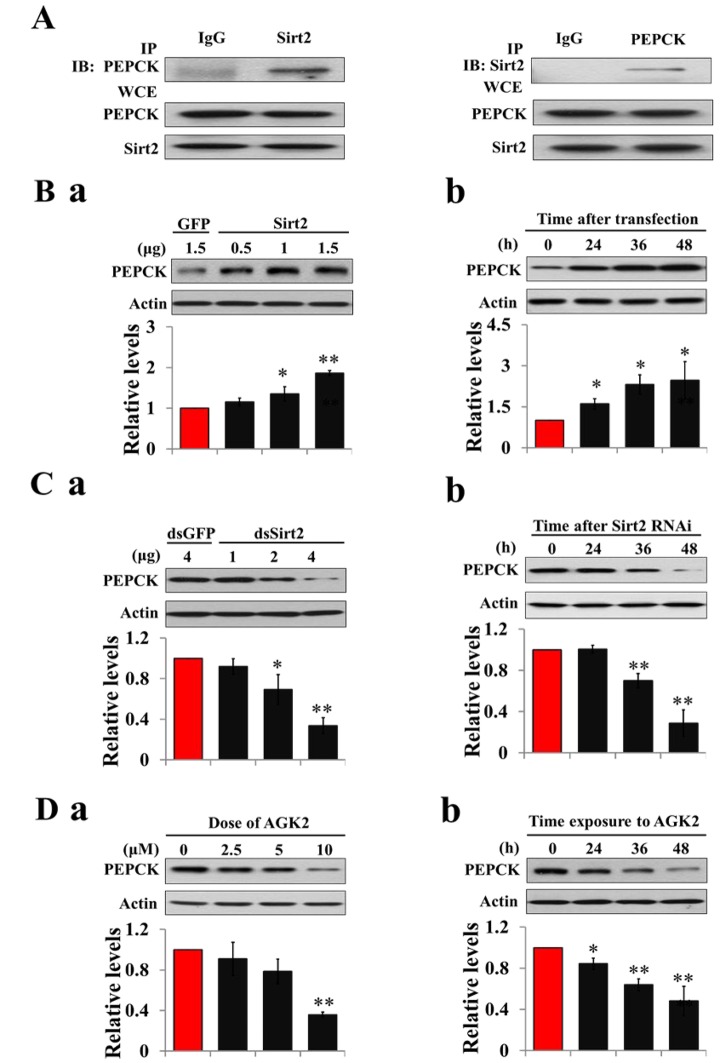
**Sirt2 interacts with and stabilizes PEPCK levels.** (**A**) Sirt2 directly binds to PEPCK. HzAm1 cells were co-transfected with GFP-Sirt2 and GFP-PEPCK-V5 plasmids for 48 h, and the cell extracts were immunoprecipitated (IP) with anti-V5 or anti-Sirt2 antibody, followed by immunoblotting (IB) with anti-Sirt2 or anti-V5 antibody, respectively. WCE: whole cell extracts. (**B**) Sirt2 transfection increases endogenous PEPCK protein levels *in vitro*. (**a**) Dose-dependent response to Sirt2 overexpression. HzAm1 cells were transfected with GFP-Sirt2 or GFP-V5 plasmid for 48 h. (**b**) Time-dependent response to Sirt2 transfection. HzAm1 cells were transfected with 1.5 μg of recombinant Sirt2 plasmid. (**C**) Sirt2 knockdown decreases endogenous PEPCK protein levels *in vitro*. (**a**) Dose-dependent response to Sirt2 RNAi. HzAm cells were transfected with Sirt2 or GFP dsRNA for 48 h. (**b**) Time-dependent response to RNAi. HzAm1 cells were transfected with 4 μg Sirt2 dsRNA. (**D**) Effects of Sirt2 inhibitor AGK2 on PEPCK protein levels. (**a**) Dose-dependent response to AGK2 treatment. HzAm1 cells were cultured with AGK2 for 48 h. (**b**) Time-dependent response to AGK2 treatment. HzAm cells were cultured with 10 μM AGK2. Proteins were extracted from the cells for IB with anti-PEPCK antibody. Protein bands were quantified using ImageJ software and normalized to the levels of *H. armigera* actin (5 μg). Each point represents the means±S.D. of three independent replicates. *, p<0.05; **, p<0.01 (determined by independent t-test).

Co-immunoprecipitation revealed that Sirt2 and PEPCK specifically bind to each other *in vitro* (Fig. 5A), suggesting a potential role for Sirt2 in PEPCK deacetylation. Moreover, an increase in endogenous PEPCK protein levels was observed by the overexpression of Sirt2 (Fig. 5B). Knockdown of Sirt2 by RNAi could also decrease protein levels of PEPCK in HzAm1 cells (Fig. 5C). To further strengthen this idea, we treated cells with Sirt2 inhibitor AGK2, and PEPCK protein levels decreased in dose- and time-dependent manners (Fig. 5D). These results suggest that Sirt2 directly binds to and stabilizes PEPCK activity.

### Acetylation regulates pyruvate homeostasis and lifespan extension

**Figure 6:**
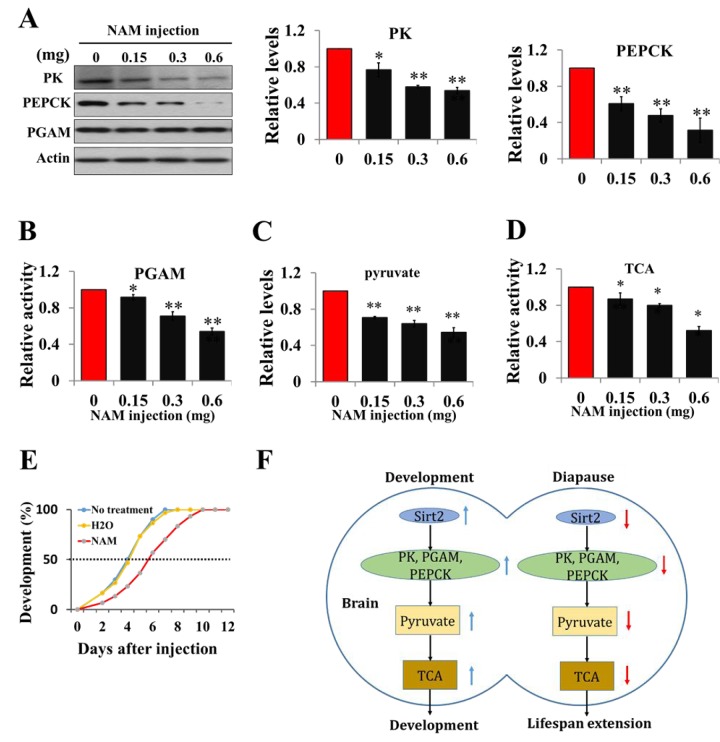
**Acetylation negatively regulates pyruvate homeostasis to lifespan extension and a schematic of the regulation of development or diapause by Sirt2-mediated changes in metabolic activity.** (**A**) Protein levels of PK, PEPCK, and PGAM in response to an injection of Sirt2 inhibitor NAM *in vivo*. Day-1 nondiapause-destined pupae were injected with NAM and the brains were dissected 48 h after injection. Proteins from pupal brains were extracted and detected with corresponding antibodies. Protein bands were quantified using ImageJ software and normalized to the levels of *H. armigera* actin (5 μg). (**B**) PGAM activity in response to an injection of NAM. (**C**) Changes of pyruvate levels in response to an injection of Sirt2 inhibitor NAM. (**D**) Changes of TCA cycle activity in response to an injection of inhibitor NAM. Day-1 nondiapause-destined pupae were injected with NAM and the brains were dissected 48 h after injection. Proteins from pupal brains were extracted. Pyruvate levels and enzymes activity was measured and normalized against total protein levels. (**E**) Developmental delay caused by NAM injection. Day-1 nondiapause-destined pupae were injected with NAM (0.6 mg). No treatment, n=60; H_2_O, n=60; NAM, n=60. Developmental delay was determined by examining the location of the pupal stemmata. Each point represents the means±S.D. of three independent replicates. *, p<0.05; **, p<0.01 (determined by independent t-test). (**F**) In nondiapause-destined pupal brain, high Sirt2 levels caused high levels of PK, PEPCK and high PGAM activity, which stimulated pyruvate generation, leading to high TCA cycle activity for development, whereas in diapause-destined individuals, low Sirt2 levels decreased the protein levels and activities of metabolic enzymes, resulting in reduced pyruvate synthesis, low metabolic activity, and induced development arrest.

To elucidate the effect of acetylation on pyruvate homeostasis *in vitro*, day-1 nondiapause-destined pupae were injected with the deacetylation inhibitor NAM, and both protein levels of PK and PEPCK and PGAM activity significantly decreased, while PGAM protein levels and *PK*, *PEPCK* mRNA levels remained unchanged (Figs. 6A-B and Fig. S8), implying that PK, PGAM, and PEPCK are regulated by acetylation in diapause pupae. Pyruvate levels and TCA cycle activity significantly decreased following NAM injection (Figs. 6C and 6D), and developmental delay for approximately 2 days was observed (Fig. 6E). These results suggest that the expression or activity of the three enzymes related to pyruvate biosynthesis is modulated by Sirt2 deacetylation, and pyruvate is a key regulator for insect diapause via the control of metabolic activity.

## DISCUSSION

Insect brain is a center for regulating diapause, and down regulation of brain activity in diapause-destined individuals leads to a decreased activity in other tissues or organs to cause diapause initiation [33]. Therefore, metabolic depression is a universal character of diapause among many species [34–36], and the most important function of sugar metabolism is related to energy production. Pyruvate is derived from glycolysis and subsequently converted into acetyl-coenzyme A to feed the TCA cycle. In the present study, low levels of pyruvate induce diapause initiation, whereas high levels of pyruvate promote pupal-adult development, indicating that pyruvate is a key regulator in development or diapause.

Based on our knowledge, PK may be the most important enzyme in the regulation of pyruvate homeostasis, as PK is the final rate-limiting enzyme to transfer a phosphate group from phosphoenolpyruvate to ADP to produce pyruvate and ATP in glycolysis. Thus, PK is directly linked to pyruvate synthesis. PGAM catalyzes the reversible conversion of 3-phosphoglycerate to 2-phosphoglycerate, which is a precursor of phosphoenolpyruvate. PEPCK is an enzyme that catalyzes oxaloacetate into phosphoenolpyruvate. PGAM and PEPCK activities are indirectly related to pyruvate synthesis. We uncovered a biochemical mechanism for how Sirt2 controls metabolic activity to extend lifespan through the deacetylation of these three metabolic enzymes related to pyruvate homeostasis.

### Sirt2 is a negative regulator to extend lifespan via the down-regulation of metabolic activity

Sirtuin is a well-known regulator of lifespan extension. Sir2 is required for longevity engendered by calorie restriction among many species [37–39]. Overexpression of Sir2 extends lifespan in budding yeast [19], and elevated sirtuin levels increase lifespan by activating DAF-16 in *C. elegans* [40], indicating a conserved role for Sir2 in lifespan extension. However, little is known about the effects of Sirt2, a homolog of Sir2 in mammals and insects, on lifespan extension. The overexpression of Sirt2 can increase lifespan by inducing the checkpoint kinase BubR1 in mice [24]. Interestingly, we observed that Sirt2 has a negative effect on lifespan via increasing the activity of metabolic enzymes in *H. armigera*, since (1) Sirt2 protein levels are higher in the brains of nondiapause-destined pupae than in those of diapause-destined pupae, consistent with high metabolic activity during pupal development; (2) overexpression of Sirt2 increases the activities of three metabolic enzymes PK, PEPCK, and PGAM related to pyruvate biosynthesis and decreases the three enzyme activities by dsRNA against Sirt2; and (3) injection of Sirt2 inhibitor into nondiapause pupae causes developmental delay (lifespan extension) through reduction in pyruvate levels and TCA cycle activity. These results suggest that low Sirt2 levels in diapause-destined pupae are associated with lifespan extension via decreasing metabolic enzyme activity and pyruvate contents, and Sirt2 is a negative regulator to extend lifespan.

### Protein deacetylation by Sirt2 is conserved in insects but has different functions

Acetylated metabolic enzymes have been identified in various tissues among different species [17,41,42]. We suspected that acetylation of metabolic enzymes in *H. armigera* might happen in the brain or other tissues. Protein acetylation is a major post-translational modification involving the reversible acetylation of the ε-amino group of internal lysine residues, which is regulated by acetyltransferases and lysine deacetylases [15,43]. Most enzymes in glucose metabolism and the TCA cycle are acetylated in mammals [44]. In the present study, we found that the acetylation of metabolic enzymes is a conserved post-translational modification to regulate pupal development in *H. armigera*. PK, PGAM, and PEPCK can be acetylated, and acetylation results in a significant reduction of the protein levels of PK and PEPCK, and PGAM activity without changes at the mRNA level. Protein acetylation has versatile effect on different enzymes [45]. It has been reported that the acetylation of PK promotes protein degradation by targeting to lysosomal [27], while the acetylation of PEPCK promotes protein degradation via proteasome in mammals [26]. However, the acetylation of PGAM caused a steric hindrance to the binding with its substrate and thereby inhibiting PGAM activity [31]. Considering the fact that the amino acid sequences of *H. armigera* PK, PEPCK and PGAM around the acetylated site are highly conservative compared with their homologs in mammals, we suspect that protein acetylation in *H. armigera* has a similar effect on protein degradation or activity as in mammals.

Although PGAM and PEPCK have been identified as substrates of Sirt2 in mammals [26,31], we also identified PK, a key enzyme for regulating pyruvate biosynthesis, as a direct target of Sirt2. Co-IP showed that Sirt2 directly binds to PK or PGAM or PEPCK and increases the deacetylation of these proteins. Sirt2 inhibitor leads to the down-regulation of the expression or activity of these three enzymes *in vitro* and *in vivo*. The results indicate that protein deacetylation by Sirt2 is conserved in insects. We have analyzed the structural characters of the three enzymes and did not find an identical or similar domain that Sirt2 binds to. The mechanism of NAD^+^-dependent deacetylation has been reported [46,47]. Upon substrate binding, the main chain of the protein substrate surrounding the acetyl-lysine makes β-sheet to contact with the catalytic domain of the sirtuin protein, burying the acetyl group within the active site of sirtuin protein. However, previous study showed that Sirt2 has little sequence selectivity, as each position near the acetyl-lysine can tolerate many different amino acids [48]. Detailed mechanism governs the substrate specificity of Sirt2 is unclear.

Previous studies have demonstrated that deacetylase Sir2 and Sirt families can extend lifespan in yeast, *C. elegans,* and *D. melanogaster* as anti-aging proteins [19]. Interestingly, sustained high levels of protein deacetylation, induced by high Sirt2 levels in nondiapause pupae, have an opposite effect in shortening lifespan, whereas low levels of Sirt2 in diapause-destined pupae extend lifespan via reduction in deacetylation of PK, PEPCK, and PGAM to decrease pyruvate levels. Thus, the function of protein deacetylation in insects is different from that in mammals.

### Low pyruvate levels extend lifespan in insects

Glucose is metabolized to produce pyruvate, which is then converted into acetyl-coenzyme A to feed the TCA cycle. Pioneering studies have indicated that lifespan can be prolonged by decreased glucose or pyruvate levels, calorie restriction reduces the plasma glucose concentration [49,50], and reduced uptake of nutritive calories can extend lifespan in multicellular eukaryotes [51,52]. Glucose restriction in *C. elegans* extends lifespan by promoting the formation of ROS, which induces catalase activity and increasing oxidative stress resistance [53], and glucose treatment during adulthood shortens the lifespan in *C. elegans* by increasing ROS formation [54] and inhibiting the activities of lifespan-extending transcription factors DAF-16/FoxO [55]. However, recent studies have shown that increasing pyruvate concentration can extend lifespan: supplementation with pyruvate at the larval period extended lifespan by increasing oxidative stress tolerance in a DAF-18/PTEN and Sir2.1-dependent manner [9]; supplementation with pyruvate at the young adult stage increased the lifespan by hyperactivating HIF-1 transcriptional activity in *C. elegans* [56]; the overexpression of malic enzyme, which oxidizes malate to pyruvate in the cytoplasm, during the larval period lengthens the lifespan in *Drosophila*, accompanied with increased ROS production and enhanced expression levels of ROS-scavenging enzymes [10]. We speculated that this paradox might reflect the distinct action of pyruvate at different developmental stages to reveal opposing effects on lifespan. In the present study, we injected pyruvate into day-1 diapause-destined pupae, and significantly more pupae were channeled into nondiapause than controls that did not receive pyruvate, suggesting that pyruvate is a checkpoint for development. Low TCA cycle activity has been associated with dauer in *C. elegans* [11] and lifespan in *Drosophila* [13]. After injecting pyruvate into diapausing pupae, TCA cycle activity increased and restarted pupal-adult development, suggesting that pyruvate regulates pupal development or diapause through the elevation or decrease of the TCA cycle activity. Therefore, physiologically decreased pyruvate levels in diapause individuals are associated with lifespan extension via a low energy metabolism.

In summary, the present results indicate that diapause, a lifespan extension phenotype, has a distinct regulatory mechanism in the brain as shown in Figure 6F. In developing individuals, high Sirt2 levels cause high levels of protein deacetylation, and metabolic enzymes PK, PEPCK, and PGAM maintain high expression or high activity. High levels of pyruvate lead to direct pupal-adult development by high metabolic activity. In contrast, low Sirt2 levels in the brains of diapause-destined pupae result in low activities of PK, PEPCK, and PGAM via decreasing protein deacetylation. Low activities of metabolic enzymes cause low levels of pyruvate production and low TCA cycle activity, finally leading to low metabolic activity for diapause initiation or lifespan extension.

## MATERIALS AND METHODS

### Insect

*H. armigera* were reared on an artificial diet at 20°C under a photoperiod of L14:D10 (light: dark) to generate nondiapause pupae, and a photoperiod of L10:D14 photoperiod to generate diapause pupae. The developmental stages were synchronized by collecting new pupae. Pupal brains were dissected in ice-cold insect saline containing 0.75% NaCl and stored at -80°C until use.

### Cell culture and transfection

HzAm1 cells were derived from the *Helicoverpa zea*, a species closely related to *H. armigera*, and cultured at 27 °C in Grace’s Insect Cell Culture Medium (GIBCO^TM^, USA) supplemented with 10% fetal bovine serum (HyClone, USA). Transfection was performed with the FuGENE^®^ HD Transfection Reagent (Promega, USA) according to the manufacturer’s instructions. The cells at log phase were suspended and plated onto 24-well plates and cultured without antibiotics. For every well of a 24-well plate, recombinant plasmid (1 μg) and transfection reagent (3 μl) were mixed in sterile water (25 μl final volume). After incubation at room temperature for 15 min, the DNA-lipid mixture was added onto the cells dropwise. Each transfection was repeated three times.

### RNA extraction, DNA amplification, and rapid amplification of cDNA ends (RACE)

Total RNA was extracted from pupal brains as described in Chen and Xu [57]. Total RNA (1 μg) was reverse transcribed at 37 °C for 1 h using M-MLV reverse transcription system (Promega, USA). Reverse transcription product (1 μl) was added to 20 μl of the PCR reaction system, and amplification was performed with degenerate primers designed according to the gene sequences of closely related species (Supplementary Table S1).

### Construction of overexpression plasmids

Full-length Sirt2, PEPCK, PK, and PGAM were amplified with primers containing the corresponding restriction sites listed in Supplementary Table S1. PCR products were digested and inserted into the plasmid GFP-piz/V5.

### *In vitro* RNA knockdown

DsRNAs targeting Sirt2 and GFP were synthesized using the T7 RiboMAX express RNAi system (Promega, USA). Primers (see Supplementary Table S1) containing T7 promoters at the 5’ ends were used to amplify the Sirt2 and GFP coding regions (400 and 700 bp, respectively). Recombinant plasmids containing Sirt2 or GFP sequences were used as templates for PCR amplification. DsRNA transfections were performed using the FuGENE transfection reagent as described above, and the cells were collected at 24-48 h after transfection. DsGFP served as a negative control.

### Protein extraction and western blot analysis

Brains and HzAm1 cells were homogenized in NP40 cell lysis buffer (150 mM NaCl, 1.0% Nonidet P-40, 50 mM Tris-HCl (pH 8.0), 1 mM PMSF, 1 mM EGTA, 5 mM NaF, and 10 mM Na_3_VO_4_). The lysate was shaken in a rotary shaker for 1 h at 4 °C, followed by centrifugation for 20 min at 12,000×*g* at 4 °C. Equal amounts of protein (20 μg for Sirt2 and PGAM, 15 μg for PEPCK, 25 μg for PK) were separated on a 10% SDS-PAGE gel and transferred to a PVDF membrane.

The synthetic peptides, corresponding to the amino acid sequences of PK, PEPCK, and PGAM in humans, were used as antigens to produce polyclonal antibodies (PK, ab137791; PEPCK, ab28455; PGAM, sc-292579). These synthetic peptide sequences showed an identity to those of *H. armigera* (Figs. S1-S3), and antibodies were purchased to investigate the expression of these three gene in insects by western blotting. The antibody against acetyl-lysine (2465195) was purchased from Millipore. Polyclonal antibodies against *H. armigera* Sirt2 and actin were generated by corresponding recombinant proteins as described in Chen and Xu [57].

### Pyruvate assay

Brains and HzAm1 cells were homogenized with 200 μl PBS and heated for 5 min at 100 °C, followed by centrifugation at 12000 ×*g* for 10 min at 4 °C. The resulting supernatant (10 μl) was added to 20 μl of 1 mM DNPH and 90 μl 50 mM sodium phosphate buffer (pH 7.0). The mixture was incubated at room temperature for 5 min. Eighty microliters of 0.6 M NaOH was added to terminate the reaction, and absorbance at 450 nm was measured by spectrophotometry.

### Citrate synthase activity

Protein (10 μg) from pupal brains or cells were incubated with the buffer (pH 8.0) containing 50 mM Tris-HCl, 10 mM KCl, 0.31 mM acetyl-CoA, 0.1 mM DTNB, and 0.5 mM oxaloacetate. Activity was measured by the change of absorbance at 412 nm with a spectrophotometer.

### PGAM activity assay

Protein (10 μg) from pupal brains or cells was incubated with the buffer containing 79 mM triethanolamine, 0.70 mM ADP, 0.15 mM NADH, 6.6 mM 3-phosphoglycerate, 1.3 mM 2,3-diphosphoglycerate, 2.5 mM MgSO_4_, 99 mM KCl, 4 units pyruvate kinase, 20 units L-lactate dehydrogenase, and 3 units enolase. Activity was measured by the change of absorbance at 340 nm resulting from NADH oxidation. All regents described above were purchased from Sigma.

### Co-immunoprecipitation and immunoblot analysis

HzAm1 cells were lysed in NP-40 cell lysis buffer, and total protein extracts (1 mg) were used for co-immunoprecipitation. The co-immunoprecipitation systems contained 25 μl of Protein G plus/Protein A-agarose suspension (Merck, USA) and 1 μg V5-tag antibody (Millipore, USA) or 1 μg Sirt2 antibody. The same amount of normal rabbit serum was used instead of antibodies as a negative control. Immunoblotting was performed with the corresponding antibodies, followed by incubation with Clean-blot IP (Thermo, USA) 1:1000, and subsequently, the blot was exposed to film.

## Supplementary Material

Supplementary File
