# Supplementary material for: Deacetylation of metabolic enzymes by Sirt2 modulates pyruvate homeostasis to extend insect lifespan
Source: Aging (Albany NY). 2018 May 16;10(5):1053–72. doi: 10.18632/aging.101447 (PMC5990394; doi:10.18632/aging.101447)
Supplement: Supplementary File [file aging-10-101447-s001.pdf]

SUPPLEMENTARY MATERIAL

Table S1.

Gene-Specific primers

**Degenerate primers**		
Sirt2-F1 CCMGACTTCCGTAGTCC	Sirt2-F2 AACCACAGGCKATATT	Sirt2-R1 TCCCCCCATCCSAGTCT
Sirt2-R2 CCSGCCTTCTCACGGTT	Sirt2-F3 GAATTATTTCCAGGAAG	
PGAM-F1 TGTKATGATYCGTCATGG	PGAM-F2 GAATGGAAYCAGAARAA	
PGAM-F3 GCWGAAGGCTATCAGTT	PGAM-R2 AACATTGTTCCAGTAWGG	
PGAM-R1 GGCAGRTTCAAYTCCAT		
PK-F1 ATCTGTACYATYGGACC	PK-F2 GTGCTVGAGAAGATGAT	
PK-R1 GGGTASTCGCCCTTGGC	PK-R2 ATACCCAGATCATCCACG	
PK-R3 TTGATGTTCTTBCCTTC		
PEPCK-F1 GAAGGATGGCTSGCCGA	PEPCK-F2 GACGACATMGCCTGGATG	
PEPCK-R1 CGGAACCAGTTBACGTG	PEPCK-R2 GCCGAAGTTGTAGCCGAA	
PEPCK-R3GTGGCCTCGSWCCTCAT		
**Overexpression primers**		
Sirt2 F CCGGAATTCTATGTCTGCAAATTCACCGCCAGG		
Sirt2 R CCGCTCGAGCGTCATAGTTCGGGCTCCTGTGG		
PK F CGGGATCCATGGTGTATACAACTATTTACGAT		
PK R CCGGAATTCCATTCCGCTTGGATGACACGCATGGT		
PGAM F CGGGATCCACATGGGACGTCAAAAGGAAAATT		
PGAM R CCCTCGAGCGCTTGGCCTTGCCTTGGGAAGCAAC		
PEPCK F CCGGAATTCCATGTTGCACCTGCAGGCTGACC		
PEPCK R CCGCTCGAGGGTGACTGTTGGACATTTTTCCTAAG		
**Prokaryotic expression primers**		
Sirt2 F CCGGAATTCGTGGAGGCTCACGGCACCTTC		
Sirt2 R CCGCTCGAGGCTCCTGTGGCGTGACGTG		
**RNA interference primers**		
Sirt2-Ri-F1 GGATCCTAATACGACTCACTATAGGGTGTTCTTCGGCGAGAGTCTC		
Sirt2-Ri-F2 GTG TTCTTCGGCGAGAGTCTC		
Sirt2-Ri-R1 GGATCCTAATACGACTCACTATAGGTAGTTC GGG CTCCTGTGGCGT		
Sirt2-Ri-R2 TAGTTCGGGCTCCTGTGGCGT		
GFP-Ri-F1: GGATCCTAATACGACTCACTATAGGAAGGGCGAGGAGCTGTTCACCG		
GFP-Ri-F2: AAGGGCGAGGAGCTGTTCACCG		
GFP-Ri-R1: GGATCCTAATACGACTCACTATAGGCAGCAGGACCATGTGATCGCGC		
GFP-Ri-F2: CAGCAGGACCATGTGATCGCGC		

Primers used for PCR in this study. M=A/C; R=A/G; W=A/T; S=G/C; Y=C/T; K=G/T; V=A/G/C; H=A/T/C; B=G/T/C; D=G/A/T.

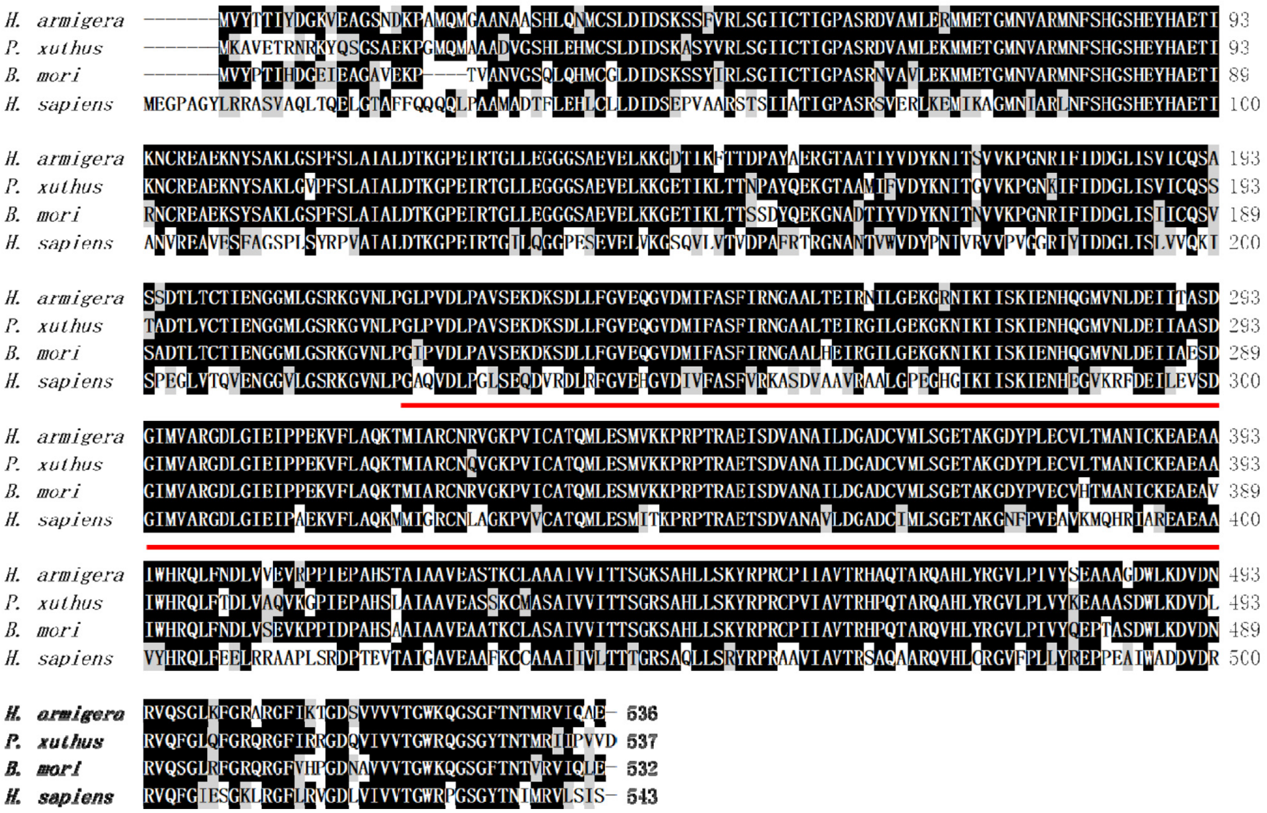

Figure S1. Homology comparison to other known PK proteins. The *H. armigera* PK amino acid sequence has high identity with PKs of other species: *P. xuthus* (89%), *B. mori* (87%), and *H. sapiens* (57%). Black shading represents ≥50% sequence identity. *H. armigera*, GenBank™ number KY412773; *P. xuthus*, XP_013164662.1; *B. mori*, XP_004931176.2; *H. sapiens*, AY352517.1. The red line below the amino acid sequence shows peptide synthesized as an immunogen.

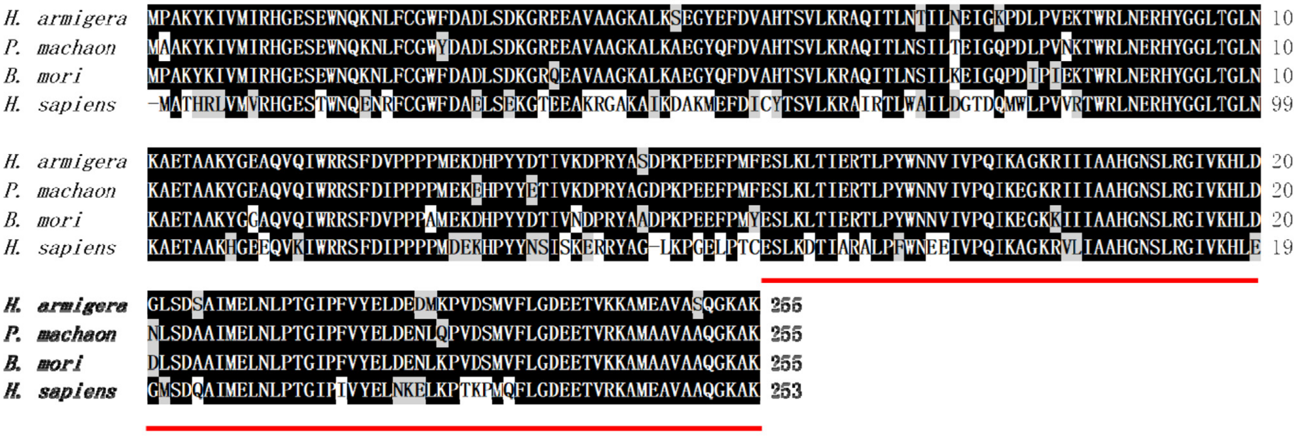

Figure S2. Homology comparison to other known PGAM proteins. The *H. armigera* PGAM amino acid sequence has high identity with PGAMs of other species: *P. machaon* (92%), *B. mori* (92%), and *H. sapiens* (69%). Black shading represents ≥50% sequence identity. *H. armigera*, GenBank™ number KY387804; *P. machaon*, XP_014361246.1; *B. mori*, NP_001037540.1; *H. sapiens*, NP_000281.2. The red line below the amino acid sequence shows peptide synthesized as an immunogen.

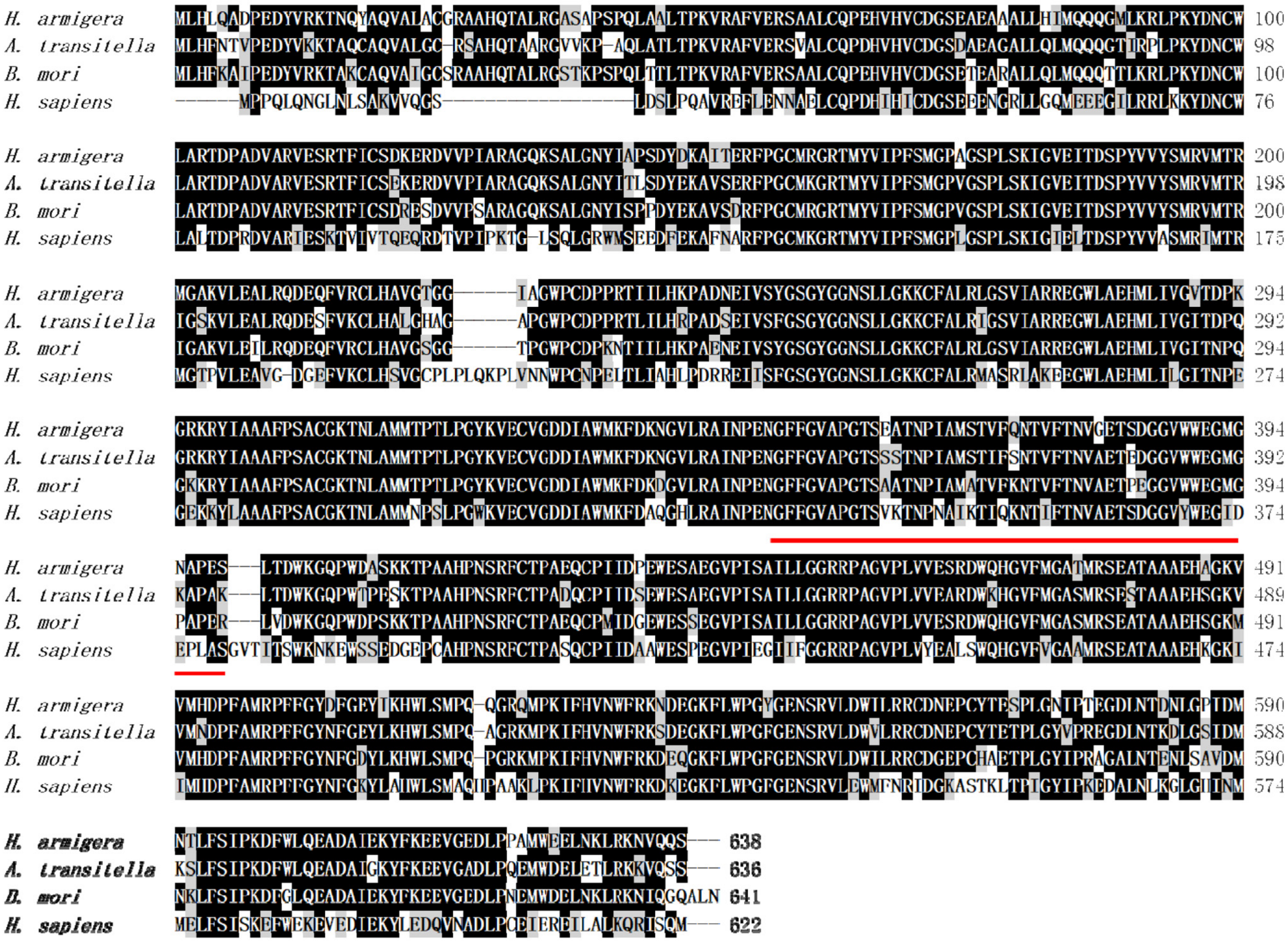

Figure S3. Homology comparison to other known PEPCK proteins. The *H. armigera* PEPCK amino acid sequence has high identity with PEPCKs of other species: *A. transitella* (85%), *B. mori* (86%), and *H. sapiens* (59%). Black shading represents ≥50% sequence identity. *H. armigera*, GenBank^{TM} number AFK28502.1; *A. transitella*, XP_013191765.1; *B. mori*, NP_001040542.1; *H. sapiens*, NP_002582.3. The red line below the amino acid sequence shows peptide synthesized as an immunogen.

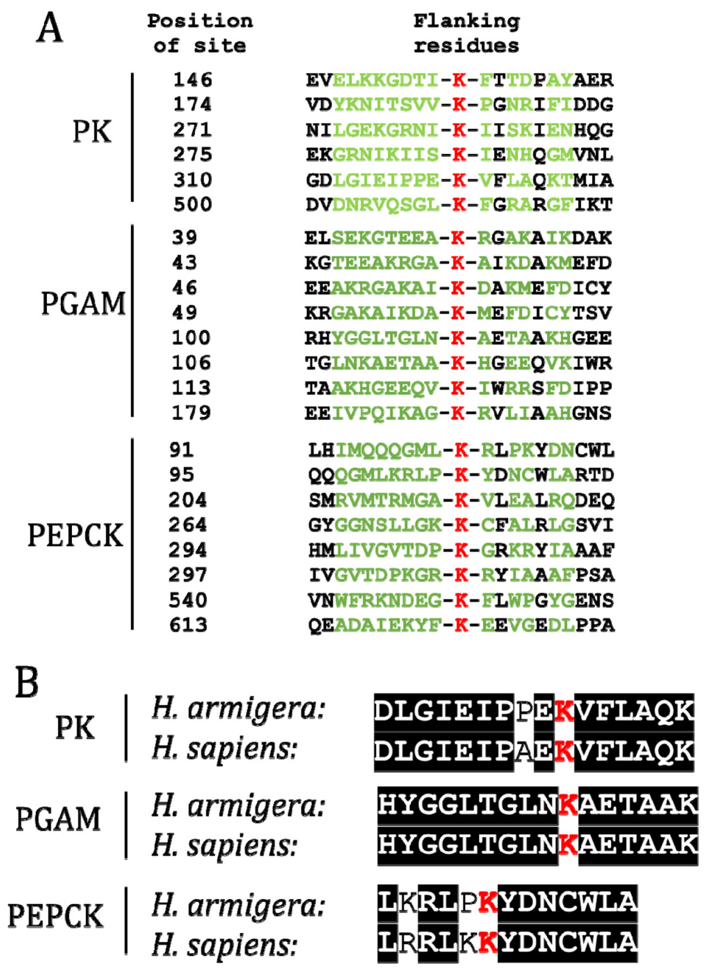

Figure S4. Predicted acetylation site of the metabolic enzymes. (**A**) Potential acetylation sites of PK, PGAM, and PEPCK using a PSKAcePred software. (**B**) Predicted acetylation sites of PK, PGAM, and PEPCK compared with known acetylation sites. The red amino acids show the predicted acetylation sites and the green amino acids show the flanking residues. *H. sapiens*, *Homo sapiens*.

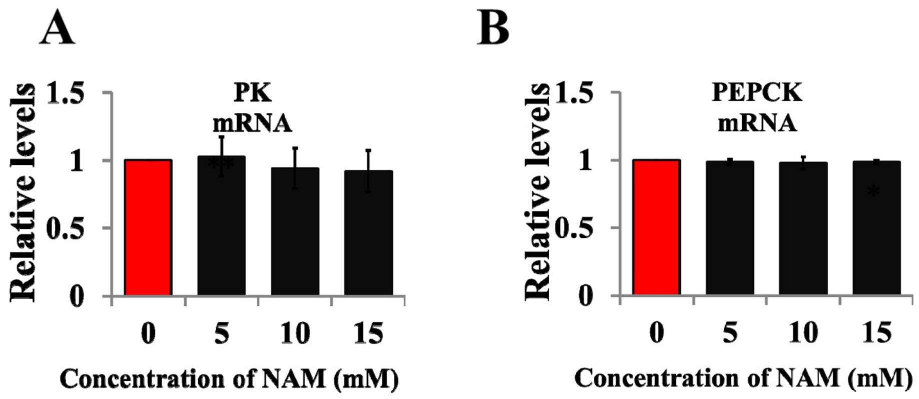

Figure S5. Effect of NAM treatment on *PK* and *PEPCK* mRNA levels. HzAm1 cells were treated with 0, 5, 10,15 mM NAM for 48 h. Total RNA was extracted and *PK* and *PEPCK* mRNA levels were detected by qPCR using *actin* as an internal standard. Each point represents the means±S.D. of three independent replicates. *, p<0.05; **, p<0.01 (determined by and independent t-test).

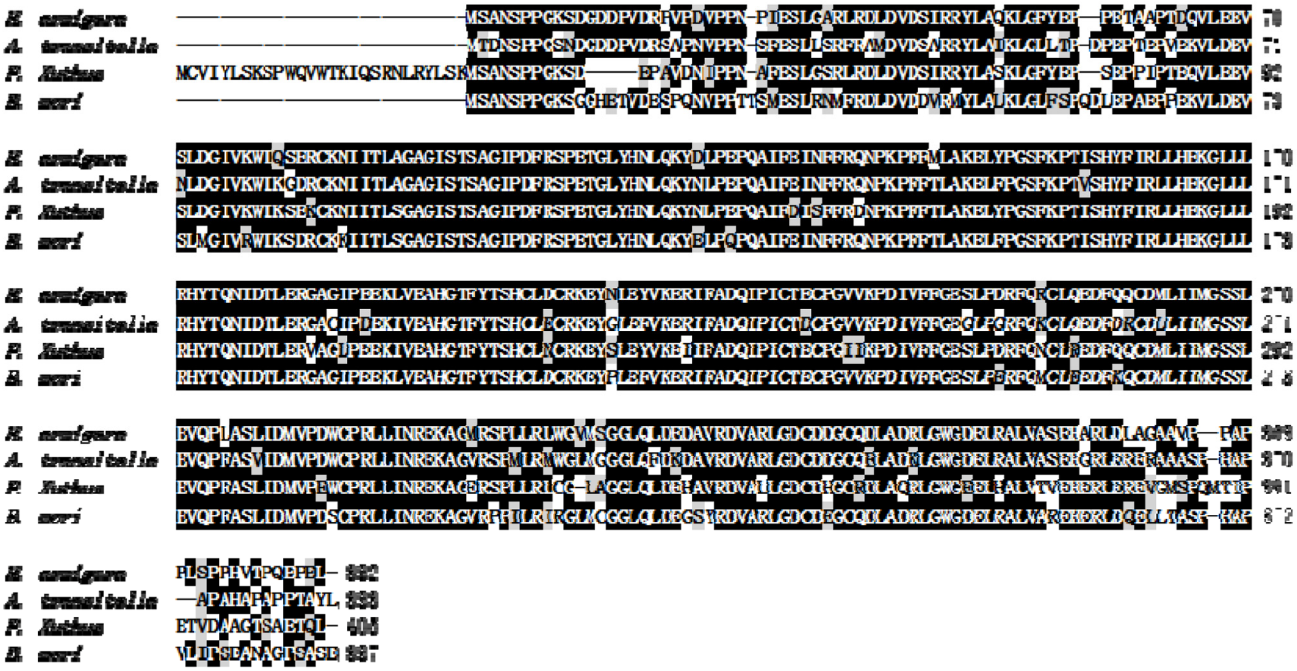

Figure S6. Homology comparison to other known Sirt2 proteins. The *H. armigera* Sirt2 amino acid sequence has high identity with Sirt2s of other species: *A. transitella* (83%), *P. xuthus* (84%), and *B. mori* (81%). Black shading represents ≥50% sequence identity. *H. armigera*, GenBank™ number KY363351; *A. transitella*, XP_013184462.1; *P. xuthus*, XP_013171078.1; *B. mori*, NP_001036937.1.

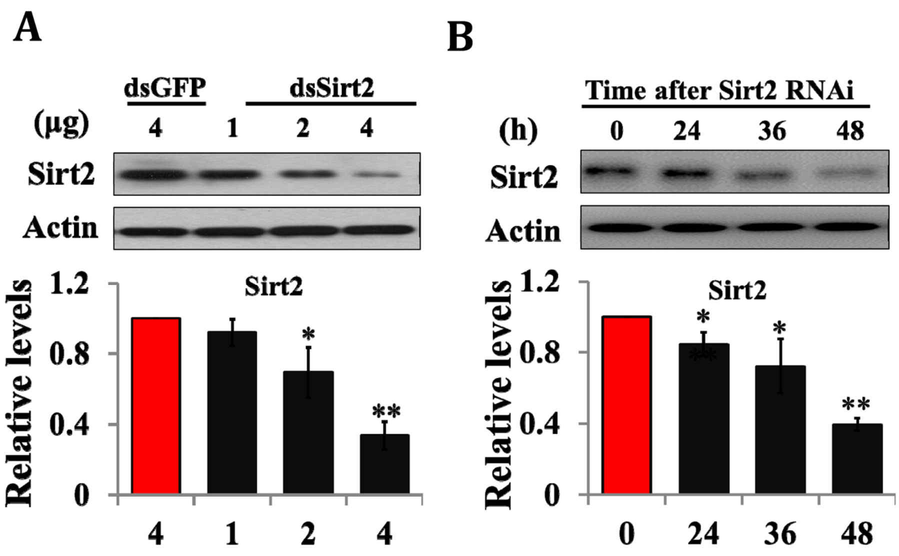

Figure S7. Efficiency of Sirt2 knockdown. (**A**) HzAm1 cells were transfected with 1, 2, and 4 μg Sirt2 dsRNA or 4 μg GFP dsRNA for 48 h. (**B**) HzAm1 cells were transfected with 4 μg Sirt2 dsRNA for 0, 24, 36, and 48 h. Protein (20 μg for Sirt2) was extracted from the cells for immunoblotting with the anti-Sirt2 antibody. Protein bands were quantified and normalized to the levels of *H. armigera* actin (5 μg). Each point represents the means±S.D. of three independent replicates. *, p<0.05; **, p<0.01 (determined by and independent t-test).

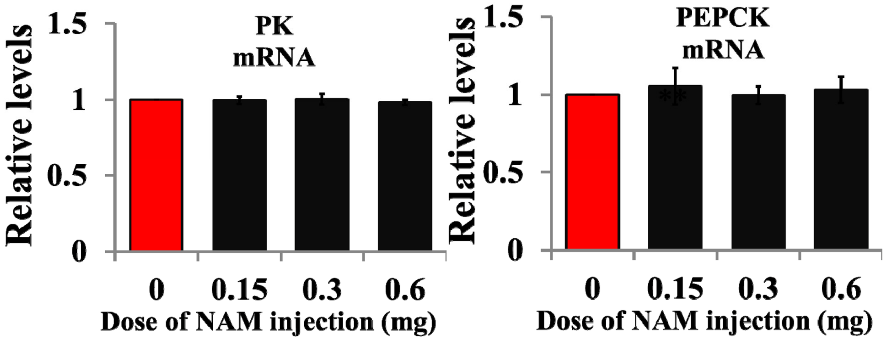

Figure S8. Effect of NAM injection on *PK* and *PEPCK* mRNA levels. Day-1 nondiapause-destined pupae were injected with NAM and pupal brains were dissected 48 hours after injection. *PK* and *PEPCK* mRNA levels were detected by qPCR using *actin* as an internal standard. Each point represents the mean±S.D. of three independent replicates. *, p<0.05; **, p<0.01 (determined by and independent t-test).
